# Supplementary material for: Generation of Fad2 and Fad3 transgenic mice that produce n-6 and n-3 polyunsaturated fatty acids
Source: Open Biol. 2019 Oct 23;9(10):190140. doi: 10.1098/rsob.190140 (PMC6833225; doi:10.1098/rsob.190140)
Supplement: Supplemental figures and tables [file rsob190140supp1.pdf]

Electronic supplementary material

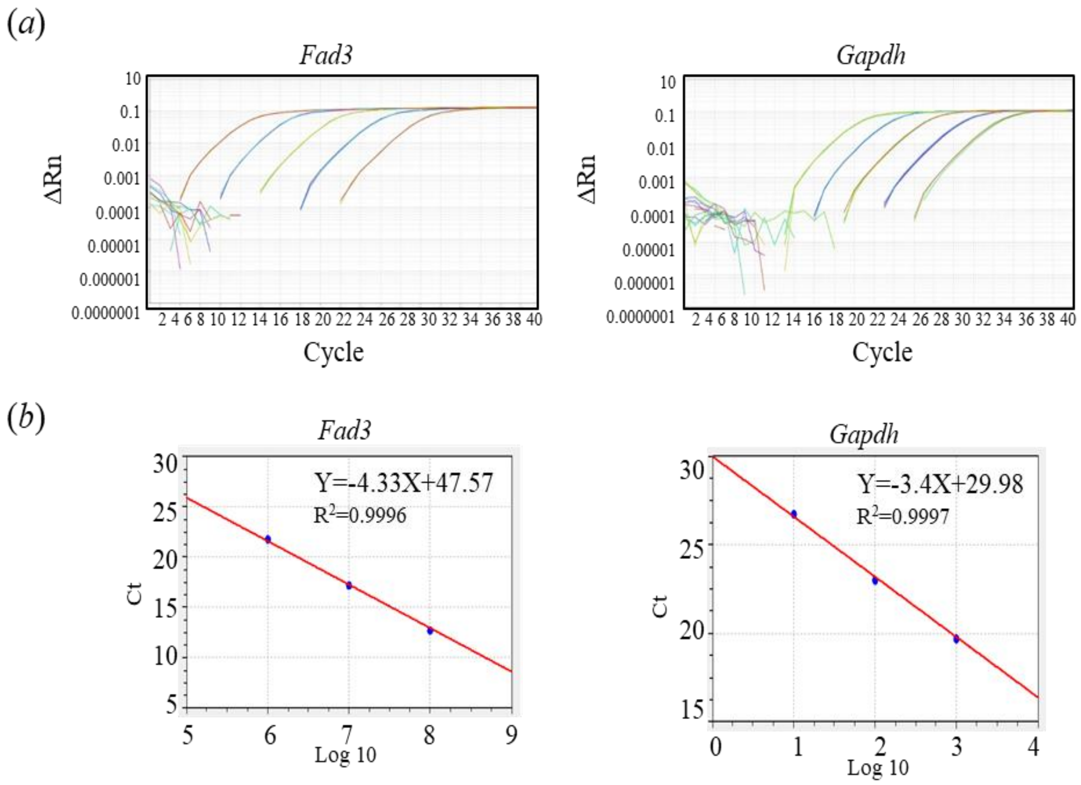

Figure S1. Standard curve of *Fad3* and *Gapdh*.

(*a*) Amplification curve of *Fad3* and *Gapdh*.

(*b*) Standard curve and equation of *Fad3* and *Gapdh*.

Table S1. Ct values obtained from the *Fad3* and *Gapdh* standards and the corresponding copies by qPCR.

Standard Samples	Mean Ct value	copies
10^5-*Fad3*	25.94	1.00×10^5
10^6-*Fad3*	21.74	1.00×10^6
10^7-*Fad3*	17.15	1.00×10^7
10^8-*Fad3*	12.70	1.00×10^8
10^9-*Fad3*	8.80	1.00×10^9
10^0-*Gapdh*	30.00	1.00×10^0
10^1-*Gapdh*	26.70	1.00×10^1
10^2-*Gapdh*	23.01	1.00×10^2
10^3-*Gapdh*	19.70	1.00×10^3
10^4-*Gapdh*	16.50	1.00×10^4

Table S2. Ct Values and the copies of *Fad3* and *Gapdh* in the *Fad3* single and *Fad2-Fad3* double transgenic mice genome.

Genotype-number		*Fad3*		*Gapdh*		The copies of transgenic mice
		Average Ct	Copies	Average Ct	Copies	
Fad3-2	♂	25.45	128394.09	16.43	9667.05	13.28
Fad3-4	♀	26.72	65349.53	17.53	4589.49	14.24
Fad3-11	♀	26.62	68918.72	15.96	13290.14	5.19
Fad2-Fad3-1	♂	23.11	445615.96	14.75	30158.64	14.78
Fad2-Fad3-3	♀	27.82	36408.34	16.65	8328.91	4.37
Fad2-Fad3-4	♀	27.74	37990.65	16.78	7626.99	4.98
Fad2-Fad3-5	♀	24.36	229233.00	15.59	17074.69	13.43
Fad2-Fad3-7	♀	29.70	13397.41	18.93	1778.28	7.53

Table S3. PUFA compositions of seven major tissues in *Fad3* F0/F1 transgenic mice and wild-type mice.

Fatty acids	Skeletal muscle			Fat			Heart			Liver			Spleen			Lung			Kidney		
	WT (n=8)	*Fad3* F0(n=3)	*Fad3* F1(n=8)	WT (n=8)	*Fad3* F0(n=3)	*Fad3* F1(n=8)	WT (n=8)	*Fad3* F0(n=3)	*Fad3* F1(n=8)	WT (n=8)	*Fad3* F0(n=3)	*Fad3* F1(n=8)	WT (n=8)	*Fad3* F0(n=3)	*Fad3* F1(n=8)	WT (n=8)	*Fad3* F0(n=3)	*Fad3* F1(n=8)	WT (n=8)	*Fad3* F0(n=3)	*Fad3* F1(n=8)
OA	24.32±2.99^{a}	24.23±2.7^{a}	25.03±3.12^{a}	28.01±3.14^{a}	27.03±2.15^{a}	26.93±4.32^{a}	16.66±1.26^{a}	14.98±0.6^{a}	15.67±0.12^{a}	17.78±1.23^{a}	16.12±2.38^{a}	15.89±1.34^{a}	13.32±0.37^{a}	12.05±1.29^{a}	11.91±2.70^{a}	12.21±1.14^{a}	11.95±0.26^{a}	12.57±3.11^{a}	14.03±1.4^{a}	13.76±2.01^{a}	15.23±2.16^{a}
LA	12.03±0.42^{a}	4.32±0.33^{b}	4.53±0.12^{b}	15.45±0.09^{a}	6.33±0.27^{b}	5.64±0.47^{b}	8.74±0.04^{a}	5.43±0.18^{b}	3.98±0.38^{c}	10.02±0.38^{a}	5.73±0.29^{b}	6.00±0.35^{b}	6.54±0.49^{a}	4.32±0.13^{b}	3.23±0.46^{c}	6.56±0.73^{c}	3.53±0.59^{b}	3.05±0.54^{b}	7.84±0.31^{a}	3.43±1.85^{b}	4.42±2.13^{b}
γ-LA	0.32±0.02^{a}	0.18±0.02^{b}	0.23±0.04^{b}	0.24±0.02^{a}	0.11±0.01^{b}	0.05±0.04^{b}	0.23±0.01^{a}	0.09±0.02^{b}	0.07±0.04^{b}	0.32±0.02^{a}	0.04±0.01^{b}	0.04±0.01^{b}	0.07±0.04^{a}	0.05±0.03^{b}	0.04±0.02^{b}	0.07±0.02^{a}	0.02±0.65^{b}	0.04±0.02^{b}	0.17±0.02^{a}	0.02±0.01^{b}	0.02±0.01^{b}
AA	1.21±0.42^{a}	0.93±0.49^{b}	0.73±0.04^{b}	2.31±0.50^{a}	1.12±0.03^{b}	1.32±0.30^{b}	1.18±0.22^{a}	0.33±0.08^{b}	0.29±0.08^{b}	1.33±0.40^{a}	0.89±0.22^{b}	0.87±0.15^{b}	0.90±0.32^{a}	0.50±0.13^{b}	0.58±0.02^{b}	2.31±0.29^{a}	1.23±0.19^{b}	1.23±0.14^{b}	1.09±0.13^{a}	0.42±0.12^{b}	0.37±0.11^{b}
ALA	0.21±0.10^{a}	0.42±0.33^{b}	0.33±0.05^{b}	0.32±0.06^{a}	0.77±0.36^{b}	0.82±0.05^{b}	0.32±0.03^{a}	0.56±0.20^{b}	0.78±0.12^{b}	0.23±0.03^{a}	0.44±0.22^{b}	0.53±0.23^{b}	0.12±0.03^{a}	0.33±0.07^{b}	0.21±0.01^{b}	0.22±0.08^{a}	0.53±0.11^{b}	0.45±0.03^{b}	0.56±0.09^{a}	1.21±0.38^{b}	1.54±0.48^{c}
EPA	0.44±0.02^{a}	0.78±0.2^{b}	0.87±0.03^{b}	0.12±0.03^{a}	0.44±0.22^{b}	0.36±0.13^{b}	0.13±0.05^{a}	0.30±0.09^{b}	0.34±0.04^{b}	0.30±0.13^{a}	0.71±0.17^{b}	0.67±0.10^{b}	0.21±0.12^{a}	0.42±0.15^{b}	0.55±0.14^{b}	0.09±0.01^{a}	0.19±0.18^{b}	0.16±0.04^{b}	0.23±0.04^{a}	0.42±0.20^{b}	0.32±0.45^{b}
DHA	4.53±0.37^{a}	10.65±1.04^{b}	8.75±0.63^{b}	7.04±0.32^{a}	12.03±0.73^{b}	11.53±1.31^{b}	3.22±0.54^{a}	8.80±0.57^{b}	9.06±1.40^{b}	5.52±0.05^{a}	10.93±0.53^{b}	8.97±1.02^{b}	2.39±0.61^{a}	4.23±0.15^{b}	5.43±0.12^{b}	3.56±0.07^{a}	6.97±0.39^{b}	9.65±1.22^{c}	3.49±0.62^{a}	6.56±0.09^{b}	5.54±0.14^{b}
n-6 total	13.56±0.86^{a}	5.43±0.84^{b}	5.49±0.20^{b}	18.00±0.61^{a}	7.56±0.31^{b}	7.01±0.81^{b}	10.15±0.27^{a}	5.85±0.28^{b}	4.34±0.50^{b}	11.67±2.30^{a}	6.66±0.52^{b}	6.91±0.51^{b}	7.51±0.85^{a}	4.87±0.29^{b}	3.85±0.50^{b}	8.94±1.04^{a}	4.78±1.43^{b}	4.32±0.80^{b}	9.10±0.46^{a}	3.87±1.98^{b}	4.81±2.25^{b}
n-3 total	5.18±0.49^{a}	11.85±1.58^{b}	9.95±0.71^{b}	7.48±0.41^{a}	13.24±1.31^{b}	12.71±1.49^{b}	3.67±0.62^{a}	9.66±0.86^{b}	10.18±1.56^{b}	6.05±0.21^{a}	12.08±0.92^{b}	10.17±1.35^{b}	2.72±0.76^{a}	4.98±0.37^{b}	6.19±0.27^{c}	3.87±0.16^{a}	7.69±0.68^{b}	10.26±1.29^{c}	4.28±0.75^{a}	8.19±0.67^{b}	7.40±1.07^{b}

WT: wild-type. Data are expressed as means ± S.D.; different superscript letters (a-c) represent statistical significant differences ($P < 0.05$).

Table S4. PUFA compositions of seven major tissues in *Fad2-Fad3* F0/F1 transgenic mice and wild-type mice.

**Fatty acids**	**Skeletal muscle**			**Fat**			**Heart**			**Liver**			**Spleen**			**Lung**			**Kidney**		
	**WT (n=8)**	***Fad2-Fad3* F0(n=5)**	***Fad2-Fad3* F1(n=8)**	**WT (n=8)**	***Fad2-Fad3* F0(n=5)**	***Fad2-Fad3* F1(n=8)**	**WT (n=8)**	***Fad2-Fad3* F0(n=5)**	***Fad2-Fad3* F1(n=8)**	**WT (n=8)**	***Fad2-Fad3* F0(n=5)**	***Fad2-Fad3* F1(n=8)**	**WT (n=8)**	***Fad2-Fad3* F0(n=5)**	***Fad2-Fad3* F1(n=8)**	**WT (n=8)**	***Fad2-Fad3* F0(n=5)**	***Fad2-Fad3* F1(n=8)**	**WT (n=8)**	***Fad2-Fad3* F0(n=5)**	***Fad2-Fad3* F1(n=8)**
OA	24.32±2.99^{a}	12.32±1.03^{b}	13.02±1.26^{b}	28.01±3.14^{a}	10.03±1.06^{c}	12.21±1.73^{b}	16.66±1.26^{a}	10.44±1.73^{b}	8.78±0.34^{c}	17.78±1.23^{a}	8.47±2.03^{b}	8.34±0.43^{b}	13.32±0.37^{a}	6.54±2.07^{b}	7.75±2.54^{b}	12.21±1.14^{a}	4.33±0.41^{b}	3.23±0.02^{b}	14.03±1.4^{a}	10.04±0.56^{b}	8.75±0.67^{c}
LA	12.03±0.42^{a}	15.01±1.17^{b}	15.65±0.21^{b}	15.45±0.09^{a}	19.21±1.9^{b}	18.31±1.05^{b}	8.74±0.04^{a}	13.74±2.05^{b}	12.03±1.74^{b}	10.02±0.38^{a}	14.01±2.03^{b}	15.04±1.03^{b}	6.54±0.49^{a}	9.98±1.32^{b}	9.34±0.55^{b}	6.56±0.73^{a}	10.22±0.89^{b}	9.49±1.30^{b}	7.84±0.31^{a}	12.75±1.48^{b}	11.09±1.09^{b}
γ-LA	0.32±0.02^{a}	1.21±0.05^{b}	1.53±0.03^{c}	0.24±0.02^{a}	0.41±0.38^{b}	0.44±0.12^{b}	0.23±0.01^{a}	0.60±0.41^{b}	0.41±0.03^{b}	0.32±0.02^{a}	0.65±0.21^{b}	0.43±0.12^{b}	0.07±0.04^{a}	0.12±0.08^{a}	0.23±0.05^{b}	0.07±0.02^{a}	0.13±0.01^{a}	0.14±0.01^{b}	0.17±0.02^{a}	0.34±0.09^{b}	0.32±0.03^{b}
AA	1.21±0.42^{a}	2.21±0.71^{b}	1.56±0.22^{b}	2.31±0.50^{a}	3.31±0.04^{b}	3.27±0.38^{b}	1.18±0.22^{a}	2.54±0.06^{b}	2.12±0.13^{b}	1.33±0.40^{a}	2.98±0.69^{b}	2.74±0.22^{b}	0.90±0.32^{a}	1.93±0.92^{b}	2.53±0.38^{b}	2.31±0.29^{a}	3.67±0.09^{b}	3.33±0.82^{b}	1.09±0.13^{a}	1.71±0.19^{b}	1.76±0.22^{b}
ALA	0.21±0.10^{a}	1.21±0.14^{b}	0.89±0.05^{b}	0.32±0.06^{a}	0.61±0.31^{b}	0.72±0.20^{b}	0.32±0.03^{a}	0.55±0.03^{b}	0.45±0.04^{b}	0.23±0.03^{a}	1.12±0.46^{b}	1.12±0.03^{b}	0.12±0.03^{a}	0.24±0.16^{b}	0.22±0.03^{b}	0.22±0.08^{a}	0.64±0.03^{b}	0.89±0.12^{b}	0.56±0.09^{a}	1.98±0.13^{b}	1.78±0.06^{b}
EPA	0.44±0.02^{a}	2.22±0.03^{b}	1.77±0.23^{b}	0.12±0.03^{a}	0.54±0.08^{b}	0.63±0.09^{b}	0.13±0.05^{a}	0.54±0.16^{b}	0.42±0.13^{b}	0.30±0.13^{a}	1.56±0.21^{b}	1.67±0.21^{b}	0.21±0.12^{a}	0.43±0.22^{b}	2.53±0.40^{c}	0.09±0.01^{a}	0.47±0.06^{b}	0.45±0.05^{b}	0.23±0.04^{a}	0.63±0.09^{b}	0.76±0.20^{b}
DHA	4.53±0.37^{a}	17.34±1.21^{b}	16.23±1.55^{b}	7.04±0.32^{a}	19.32±0.98^{b}	21.43±1.61^{b}	3.22±0.54^{a}	15.54±0.05^{c}	11.44±2.01^{b}	5.52±0.05^{a}	15.96±0.22^{b}	17.43±1.76^{b}	2.39±0.61^{a}	8.87±0.52^{b}	9.49±0.29^{b}	3.56±0.07^{a}	12.98±1.07^{b}	10.79±0.49^{b}	3.49±0.62^{a}	12.94±1.08^{b}	15.68±0.17^{c}
n-6 total	13.56±0.86^{a}	18.43±1.93^{b}	18.74±0.46^{b}	18.00±0.61^{a}	22.93±2.32^{b}	22.02±1.55^{b}	10.15±0.27^{a}	16.88±2.52^{b}	14.56±1.90^{b}	11.67±2.30^{a}	17.64±2.93^{b}	18.21±1.37^{b}	7.51±0.85^{a}	12.03±2.32^{b}	12.10±0.98^{b}	8.94±1.04^{a}	14.02±0.99^{b}	12.96±2.13^{b}	9.10±0.46^{a}	14.8±1.76^{b}	13.17±1.34^{b}
n-3 total	5.18±0.49^{a}	20.77±1.38^{b}	18.89±1.83^{b}	7.48±0.41^{a}	20.47±1.37^{b}	22.78±1.90^{b}	3.67±0.62^{a}	16.63±0.24^{c}	12.31±2.18^{b}	6.05±0.21^{a}	18.64±0.89^{b}	20.22±2.00^{b}	2.72±0.76^{a}	9.54±0.90^{b}	12.24±0.72^{c}	3.87±0.16^{a}	14.09±1.16^{b}	12.13±0.66^{b}	4.28±0.75^{a}	15.55±1.30^{b}	18.22±0.43^{b}

WT: wild-type. Data are expressed as means ± S.D.; different superscript letters (a-c) represent statistical significant differences ($P < 0.05$).

Table S5. A tabulation of primer sequences.

Application	Gene	Sequence (5'-3')	Length
Genes amplification	*Fad3*	forward: AAAACTGTGGCTCTGCAGGA<br>reverse: CAGCCTGCATATATCAGAGC	1,233 bp
	Fad2	forward: CACACTTCGGCCTCTCCTTC<br>reverse: AAAATTCACAAATAATTGTGT	1,627 bp
Genotyping of transgenic mice	*Fad3*	forward: GTTCTGGGCAGTCTTTGTT<br>reverse: GGTACGGAACTTGGGAATA	865 bp
	Fad2	forward: GCTGGAACAATGGGTGC<br>reverse: GCCAAGGGTTAGCGTCA	573 bp
RT-PCR	*Fad3*	forward: CTCCCAATGTTTGCTTATCC<br>reverse: CGAGCCATGCCACGAAT	235 bp
	Fad2	forward: ACCATTGCGTTCCTCCTC<br>reverse: AAGCCAACAGTGTCATCG	191 bp
	Gapdh	forward: GAGTGTTTCCTCGTCCCG<br>reverse: CCGTTGAATTTGCCGTGA	193 bp
Detection of copy number	*Fad3*	forward: AGCCGAAGAAATCAGGG<br>reverse: TTGGGAATATGTGGGTCAG	127 bp
	Gapdh	forward: GGCCTCCAAGGAGTAAGAAA<br>reverse: GCCCCTCCTGTTATTATGG	141 bp
qPCR	*Fasn*	forward: GGAGGTGGTGATAGCCGGTAT<br>reverse: TGGGTAATCCATAGAGCCCAG	140 bp
	Scd1	forward: TTCTTGCGATACACTCTGGTGC<br>reverse: CGGGATTGAATGTTCTTGTCGT	98 bp
	Acc	forward: ATGGGCGGAATGGTCTCTTTC<br>reverse: TGGGGACCTTGTCTTCATCAT	148 bp
	Lipe	forward: CCAGCCTGAGGGCTTACTG<br>reverse: CTCCATTGACTGTGACATCTCG	106 bp
	Lpl	forward: GGGAGTTTGGCTCCAGAGTTT<br>reverse: TGTGTCTTCAGGGGTCCTTAG	115 bp
	PPARγ	forward: TCGCTGATGCACTGCCTATG<br>R: GAGAGGTCCACAGAGCTGATT	103 bp
	Lcad	forward: TCTTTTCCTCGGAGCATGACA<br>reverse: GACCTCTCTACTCACTTCTCCAG	113 bp
	Fabp4	forward: AAGGTGAAGAGCATCATAACCCT<br>reverse: TCACGCCTTTCATAACACATTCC	133 bp
	Gapdh	forward: GTGGCAAAGTGGAGATTGTTG<br>reverse: CTCCTGGAAGATGGTGATGG	164 bp
